# Clinical Significance of Serum Oxidative Stress Markers to Assess Disease Activity and Severity in Patients With Non-Segmental Vitiligo

**DOI:** 10.3389/fcell.2021.739413

**Published:** 2021-12-16

**Authors:** Shuli Li, Wei Dai, Sijia Wang, Pan Kang, Zhubiao Ye, Peng Han, Kang Zeng, Chunying Li

**Affiliations:** ^1^ Department of Dermatology and Venereology, Nanfang Hospital, Southern Medical University, Guangdong, China; ^2^ Department of Dermatology, Xijing Hospital, Fourth Military Medical University, Xi’an, China; ^3^ Department of Otolaryngology, First Affiliated Hospital, Xi’an Jiaotong University, Xi’an, China

**Keywords:** non-segmental vitiligo, oxidative stress markers, activity, severity, CXCL10, vitiligo subtypes

## Abstract

Non-segmental vitiligo (NSV) is a chronic autoimmune disease characterized by progressive depigmentation of the skin. Oxidative stress (OS) has been proposed as one among the main principal causes in the development and establishment of a sustained autoimmune state in patients with NSV. However, the disease-associated OS biomarkers in clinical practice are not well studied. In this study, we found significantly reduced antioxidant enzymes [catalase (CAT) and superoxide dismutase (SOD)], total antioxidant capacity (TAC), and increased levels of lipid oxidation product malondialdehyde (MDA) and oxidative DNA damage byproduct [8-hydroxy-2-deoxyguanosine (8-OHdG)] in serum of NSV patients compared with healthy controls (HC). Serum TAC, MDA, and 8-OHdG levels were correlated with disease activity in all patients with NSV and much lower in patients receiving conventional treatment in the past 1 year compared to that without treatment. In addition, both serum MDA and 8-OHdG levels were significantly correlated with CXCL10 expression in patients with NSV. And the serum TAC, MDA, and 8-OHdG levels were also correlated with affected body surface area and Vitiligo Area Scoring Index score in patients with NSV. This study demonstrates dysregulated OS status in patients with NSV and provides the evidence that the serum TAC, MDA, and 8-OHdG have a capacity to indicate the activity and severity in patients with NSV.

## Introduction

Vitiligo is a chronic autoimmune skin disease characterized by depigmented skin due to loss of epidermal melanocytes, affecting 0.5%–2% of the world population (Picardo et al., 2015). Although the exact etiology of non-segmental vitiligo (NSV) is not fully understood, studies suggest that oxidative stress (OS) plays a critical role in vitiligo initiation and development (Picardo et al., 2015). We and other groups have previously demonstrated that OS not only can lead to multiple forms of melanocyte death (Chen et al., 2021) but also can induce the secretion of damage-associated molecular patterns, such as high-mobility group protein B1 and inducible heat shock protein 70 (Mosenson et al., 2014; Cui et al., 2019), and the inflammatory cytokines, including IL-6, IL-8, CXCL12, CCL5, CXCL10, and CXCL16 from stressed melanocytes or keratinocytes (Toosi et al., 2012; Li et al., 2017; Rezk et al., 2017; Li et al., 2020), initiating or promoting the autoimmune reaction toward melanocytes. The critical role of OS in the development and establishment of an autoimmune state in vitiligo has been widely accepted; however, limited evidence is available on disease-associated OS markers that are associated with the vitiligo activity or severity.

Clinically, the evaluation of vitiligo activity and severity predominantly relies on the subjective observation of clinical features. Although the efforts to develop disease associated circulating markers are ongoing (Tsai et al., 2019), the focus is mainly on immunologic markers, such as soluble CD molecules (sCD27 and sCD25), chemokine CXCL10, and S100B (Speeckaert et al., 2016; Wang et al., 2016; Speeckaert et al., 2017). Previous studies suggest that OS is not only limited to skin lesion but also in the systemic system of the patients. Therefore, additional research is needed to identify the possible OS markers to guide the management and treatment for vitiligo.

OS is characterized by the excessive accumulation of oxidation products and inadequate antioxidants (Chen et al., 2021). There is numerous evidence that patients with vitiligo have increased oxidation product like the lipid oxidation product malondialdehyde (MDA) and the oxidative DNA damage byproducts [8-hydroxy-2-deoxyguanosine (8-OHdG)] (Wei et al., 2013; Vaseghi et al., 2017; Azzazi et al., 2021). In addition, patients with vitiligo have down-regulated enzymes, such as catalase (CAT) (Agrawal et al., 2014) and dysregulated superoxide dismutase (SOD) (Koca et al., 2004; Khan et al., 2009; Ozel Turkcu et al., 2014), and abnormal non-enzyme defense to oxidants like the total antioxidant capacity (TAC) (Hayran et al., 2021). Therefore, we speculated that these OS molecules may be disease markers of vitiligo. In this study, we detected the concentration/activity of antioxidants including CAT, SOD, and TAC and the oxidation products such as MDA and 8-OHdG in serum samples from patients with NSV and healthy controls (HC). In addition, the previously reported vitiligo activity-associated markers including CXCL10 were also detected in each patient. The correlation between these OS markers and disease activity/severity was further analyzed.

## Materials and Methods

### Patients and Samples

A total of 96 patients with NSV were diagnosed clinically and recruited from the Department of Dermatology, Xijing Hospital. In addition, 64 age-, sex-, and body mass index (BMI) –matched HC were enrolled from the visitors receiving plastic surgery (including nevus removal and/or cosmetic surgery that improving the normal appearance and/or removing signs of aging) in the department. None of the HC group had a physician-diagnosed disease or perceived frailty. The study statistical plan incorporated group sizes of 43 patients with NSV and 43 HC; this sample size was sufficient to perform the serum 8-OHdG assays with an expected standard width of interval of 0.6 and confidence level of 95%, according to the formula for calculating the sample size of a descriptive study of a continuous variable (Browner et al., 2013). Additional patients and control were incorporated into the study due to their availability. The demographic profile (including the sex, age, BMI, smoking, and drinking) of both patients and HC, and the clinical characteristics and detailed subtypes of patients with NSV are recorded in Table 1. The serum was freshly isolated from whole blood samples and then stored at −80°C until analysis. All the NSV patients and HC consented through written and informed agreement for inclusion in the study. The research protocol was designed and executed according to the principles of the Declaration of Helsinki and was approved by the ethics review board of Xijing Hospital, Fourth Military Medical University.

**TABLE 1:** Characteristics of patients with NSV and HC included in this study.

Characteristics	Patients with NSV	HC	*P-*value
Sex, n/total (%)			
Male	52/96 (54.17)	33/64 (51.56)	0.746^a^
Age (years), mean (median, IQR)			
Age at blood sampling	33.80 (32, 25.75–42)	34.17 (32.5, 26.75–41)	0.83^b^
Age of disease onset	25.39 (25, 15.5–34)		
BMI (kg/m^2^), mean (median, IQR)	22.80 (22.12, 20.57–25.08)	22.52 (22.42, 20.16–25.04)	0.592^b^
Smoking, n/total (%)	22/96 (22.92)	14/64 (21.88)	0.877^a^
Drinking, n/total (%)	37/96 (39.54)	26/64 (40.63)	0.792^a^
disease Duration (months), mean (median, IQR)	97.81 (60, 15–144)		
Subtype, n/total (%)			
Focal	4/96 (4.17)		
Mucosal	4/96 (4.17)		
Acrofacial	28/96 (29.17)		
Generalized	59/96 (61.46)		
Universal	1/96 (1.04)		
Activity, n/total (%)			
Stable	20/96 (20.83)		
Mild-moderate active	37/96 (38.54)		
Active	39/96 (40.63)		
% affected BSA, mean (median, IQR)	4.51 (0.8, 0.4–2.425)		
VASI score, mean (median, IQR)	4.07 (0.7, 0.3–2)		
Halo nevi, n/total (%)	12/96 (12.5)		
Associated comorbid autoimmune disease, n/total (%)			
Thyroid disease	2/96 (2.08)		
Psoriasis	1/96 (2.08)		
Type 1 diabetes mellitus	1/96 (1.04)		
Alopecia areata	1/96 (1.04)		
Therapy during 1-year period before blood sampling, n/total (%)			
No treatment	22/96 (22.92)		
NB-UVB phototherapy	4/96 (4.17)		
Oral Chinese medicine	5/96 (5.21)		
NB-UVB phototherapy combined with topical corticosteroid/tacrolimus/pimecrolimus	37/96 (38.54)		
Oral Chinese medicine combined with topical corticosteroid/tacrolimus/pimecrolimus	28/96 (29.17)		

Abbreviations. NSV, non-segmental vitiligo; HC, healthy controls; IQR, interquartile range; BMI, body mass index; BSA, body surface area; VASI, vitiligo area scoring index; NB-UVB, narrow bound ultraviolet B light.

a Chi-square test.

b Independent samples *t*-test.

### Assessment of Vitiligo Disease Activity

The disease activity was clinically evaluated by three experts independently according to “The Consensus on Diagnosis and Management of Vitiligo” published in Chinese Journal of Dermatology in 2018. The final disease activity assessment or scores were averaged among the experts. The evaluation considers mainly on four aspects: clinical features, Koebner phenomenon, Wood lamp examination results, and vitiligo disease activity (VIDA) score on a six-point scale.

Active vitiligo could be determined if any item of the following four was observed: 1) clinical markers, including poorly defined borders, and inflammatory signs, such as pruritus and erythema, trichromatic vitiligo, confetti-like depigmentation, and hypopigmentation; 2) Koebner phenomenon in the past 1 year; 3) poorly demarcated borders associated with hypomelanotic edging or larger hypochromia area than visual area in a Wood’s light examination; and 4) VIDA score 1 to 4. Very active vitiligo was scored in patients with clear activity markers or obvious progression (≥1% BSA) or VIDA score = 4; mild-moderate active in patients with small progression (<1% BSA) or VIDA score 1 to 3.

Stable vitiligo could be determined if at least two items were identified: 1) clinical features: white spots with clear edges or signs of re-pigmentation; 2) no Koebner phenomenon for at least 1 year; 3) white lesion with sharply clear borders, smaller than or equal to the visual area under Wood’s light; and 4) the VIDA score is 0 or −1 point.

### Assessment of Vitiligo Disease Severity

The affected body surface area (BSA) was scored to measure the vitiligo extent by using the hand units. The hand area is about 1% of the BSA. For affected BSA less than 1%, the assessment was made by referring to the knuckle and palm units. One hand unit is equal to 32 fingertip units, one palm area is equal to l8 fingertip units, and one fingertip unit accounts for 0.03% of the BSA. The Vitiligo Area Scoring Index (VASI) score system was used to offer a disease severity index (Hamzavi et al., 2004). Both the BSA and VASI scores were evaluated by three experts independently, and the final scores were averaged among the experts.

### OS Markers Detection

CAT activity was detected by CAT Assay Kit (Beyotime Biotechnology, Beijing, China) on the basis of the rate of degradation of hydrogen peroxide (H_2_O_2_) in the sample. One unit of CAT activity was defined as the amount of the enzyme that decomposes 1 μmol H_2_O_2_ per minute. The assay was carried out at a 520-nm wavelength.

SOD activity was detected by SOD Assay Kit (Beyotime Biotechnology, Beijing, China) by using the classic nitrogen blue tetrazole (NBT) method. Briefly, the Xanthine and Xanthine oxidase reaction system could produce superoxide anion (O_2_^−.^), which could be catalyzed by SOD to produce H_2_O_2_ and oxygen O_2_. In addition, the rest O_2_^−.^ in the sample could reduce the NBT to blue NBT formazan, which has a strong absorption peak at 560 nm. One unit of SOD activity was defined as the inhibition rate was 50% in the Xanthine and Xanthine oxidase reaction system.

TAC level was determined by using the TAC Assay Kit with 2,2′-azino-bis (3-ethylbenzothiazoline-6-sulfonic acid) (ABTS) method (T-AOC Assay Kit, Beyotime Biotechnology, Beijing, China). ABTS was oxidized to green ABTS^+^ by appropriate oxidant, which can be inhibited if there are existing antioxidants. The TAC of the sample was determined and calculated by measuring the absorbance of ABTS^+^ at 405 nm. The classic antioxidant 6-hydroxy-2,5,7,8-tetramethylchroman-2-carboxylic acid (Trolox) was used as a reference; thus, the TAC was presented as Trolox equivalent antioxidant capacity (TEAC).

MDA was detected by the Lipid Peroxidation MDA Assay Kit (Beyotime Biotechnology, Beijing, China). MDA in the catabolite of lipid peroxide reacted with thiobarbituric acid and produced red compound, which has a maximum absorption peak at 540 nm.

8-OHdG level was detected by the human 8-OHdG ELISA kit (4A Biotech, Beijing, China) according to the instructions of the manufacturer.

### CXCL10 Analysis

A Human CXCL10 ELISA kit (R&D Systems, USA) was used to analyze serum samples of the patients according to the instructions of the manufacturer.

### Statistical Analysis

All statistical analyses were performed using SPSS 23.0 (SPSS Science, Chicago, IL). The chi-square test and independent samples *t*-test were used to compare the differences between patients with NSV and HC. The Mann–Whitney U test and Kruskal–Wallis analysis were used for comparison of two or more than two groups, respectively. Receiver operating characteristic (ROC) analysis was performed to determine the sensitivity and specificity of OS biomarkers and the area under the ROC curve. Correlations between OS biomarkers and affected BSA, VASI score, CXCL10, and any other clinical characters were assessed using Pearson correlation analyses. All the data were presented as the median and range using charts from individual data points. In all cases, *P*-value less than 0.05 was considered to indicate statistical significance.

## Results

### Patients

In our study, we obtained serum samples from 96 patients with NSV and 64 HC subjects. The demographic profile of both patients and HC, and the detailed clinical characteristics of patients with NSV (including the disease duration, subtypes, activity, BSA, VASI scores, presence of Halo nevi, associated comorbid autoimmune disease, and therapy during one-year period) are summarized in Table 1.

### Patients With NSV Were Characterized by Reduced Antioxidative Defense and Increased Oxidative Damage

The activity of selected antioxidant enzymes including CAT and SOD in serum of patients with NSV was significantly lower than that in the HC (Table 2). The serum TAC was also significantly reduced in patients than HC (Table 2). In addition, the markers of oxidative damage to both lipids and proteins, MDA and 8-OHdG, were significantly elevated in NSV patients compared with HC (Table 2).

**TABLE 2:** Serum levels of OS markers in HC and patients with NSV.

Groups	HC (n = 64), median (IQR)	Patients with NSV (n = 96), median(IQR)	*P* value^a^	Patients with NSV			*P*-value^b^
Parameter				Stable (n = 20), median(IQR)	Mild-moderate active (n = 37), median(IQR)	Very active (n = 39), median(IQR)<	
CAT activity (μmol H_2_O_2_/min/ml)	1.907(1.629–2.305)	1.394(0.962–1.886)	<0.0001	1.464(0.879–1.903)	1.357(0.794–1.851)	1.427(0.965–1.893)	0.6768
SOD activity (units)	1.429(1.103–1.816)	1.193(0.923–1.567)	0.0011	1.153(0.919–1.469)	1.179(0.935–1.587)	1.273(0.899–1.570)	0.9478
TAC/TEAC (mmol/L)	1.091(0.876–1.293)	0.938(0.784–1.158)	0.0053	0.930(0.808–1.143)	1.010(0.822–1.266)	0.882(0.690–1.130)	0.0709
MDA (μmol/L)	1.359(0.881–2.276)	2.339(1.433–3.624)	<0.0001	1.595(0.844–2.03)	2.515(1.411–3.613)	2.884(1.499–3.885)	0.0015
8-OHdG (pg/ml)	542.7(440.2–670.2)	812.0(623.4–1007)	<0.0001	690.6(554.1–854.9)	797.0(680.6–986.3)	967.0(664.1–1080)	0.0278

Abbreviations. OS, oxidative stress; HC, healthy controls; NSV, non-segmental vitiligo; IQR, interquartile range; CAT, catalase; SOD, superoxide dismutase; TAC, total antioxidant capacity; TEAC, trolox equivalent antioxidant capacity; MDA, malondialdehyde; 8-OHdG, 8-hydroxy-2-deoxyguanosine.

a Two-tailed Mann–Whitney U test for patients with NSV, and HC.

b Kruskal-Wallis test among the three activity stages of patients with NSV.

**FIGURE 1:**
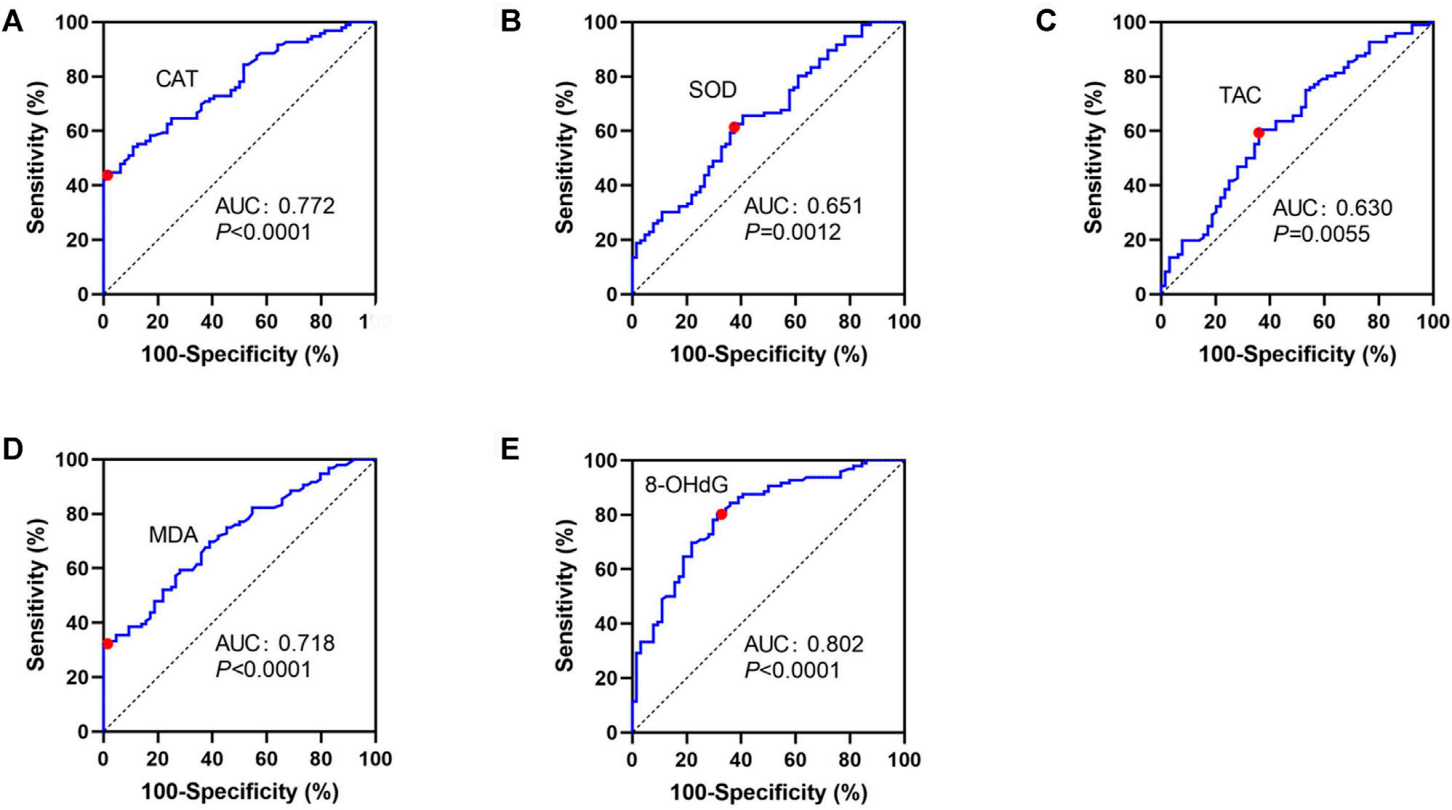
ROC curves for CAT **(A)**, SOD **(B)**, TAC **(C)**, MDA **(D)**, and 8-OHdG **(E)** in patients with NSV and HC. ROC, receiver operating characteristic curves; CAT, catalase; SOD, superoxide dismutase; TAC, total antioxidant capacity; TEAC, Trolox equivalent antioxidant capacity; MDA, malondialdehyde; 8-OHdG, 8-hydroxy-2-deoxyguanosine.

The ROC curve analysis for vitiligo diagnosis was significant for CAT, MDA, and 8-OHdG, displaying areas under the curve of 0.772, 0.718, and 0.802, respectively but not for SOD and TAC (Table 3; Figures 1A–E). For CAT, a cutoff value of 1.27 μmol H_2_O_2_/min/ml resulted in a specificity of 98.44% and a sensitivity of 44.79% (Table 3; Figure 1A). For MDA, a decided cutoff value 3.21 μmol/L showed a specificity of 100.0% and a sensitivity of 32.29% (Table 3; Figure 1D). For 8-OHdG, a cutoff set at 594.9 pg/ml displayed a specificity of 68.75% and a sensitivity of 80.21% (Table 3; Figure 1E).

**TABLE 3:** ROC analysis of OS markers in patients with NSV.

OS markers	Cutoff value	AUC (95%CI)	Sensitivity (95%CI), %	Specificity (95%CI), %	PPV, %	NPV, %
CAT activity (μmol H_2_O_2_/min/ml)	1.27	0.772(0.702–0.842)	44.79(35.24–54.75)	98.44(91.67–99.92)	97.73	54.31
SOD activity (units)	1.32	0.651(0.565–0.737)	62.50(52.51–71.53)	62.50(50.25–73.33)	71.43	52.63
TAC/TEAC (mmol/L)	0.99	0.630(0.541–0.718)	60.42(50.42–69.62)	64.06(51.82–74.71)	71.60	51.90
MDA (μmol/L)	3.21	0.718(0.640–0.796)	32.29(23.78–42.17)	100.00(94.34–100.00)	100.00	49.61
8-OHdG (pg/ml)	594.9	0.802(0.733–0.871)	80.21(71.14–86.95)	68.75(56.61–78.77)	79.38	69.84

Abbreviations. OS, oxidative stress; HC, healthy controls; NSV, non-segmental vitiligo; ROC, receiver operator characteristic; AUC, area under curve; CI, confidence interval; PPV, positive predictive value; NPV, negative predictive value; CAT, catalase; SOD, superoxide dismutase; TAC, total antioxidant capacity; TEAC, trolox equivalent antioxidant capacity; MDA, malondialdehyde; 8-OHdG, 8-hydroxy-2-deoxyguanosine.

### OS Markers Were Associated With Disease Activity in Patients With NSV

**FIGURE 2:**
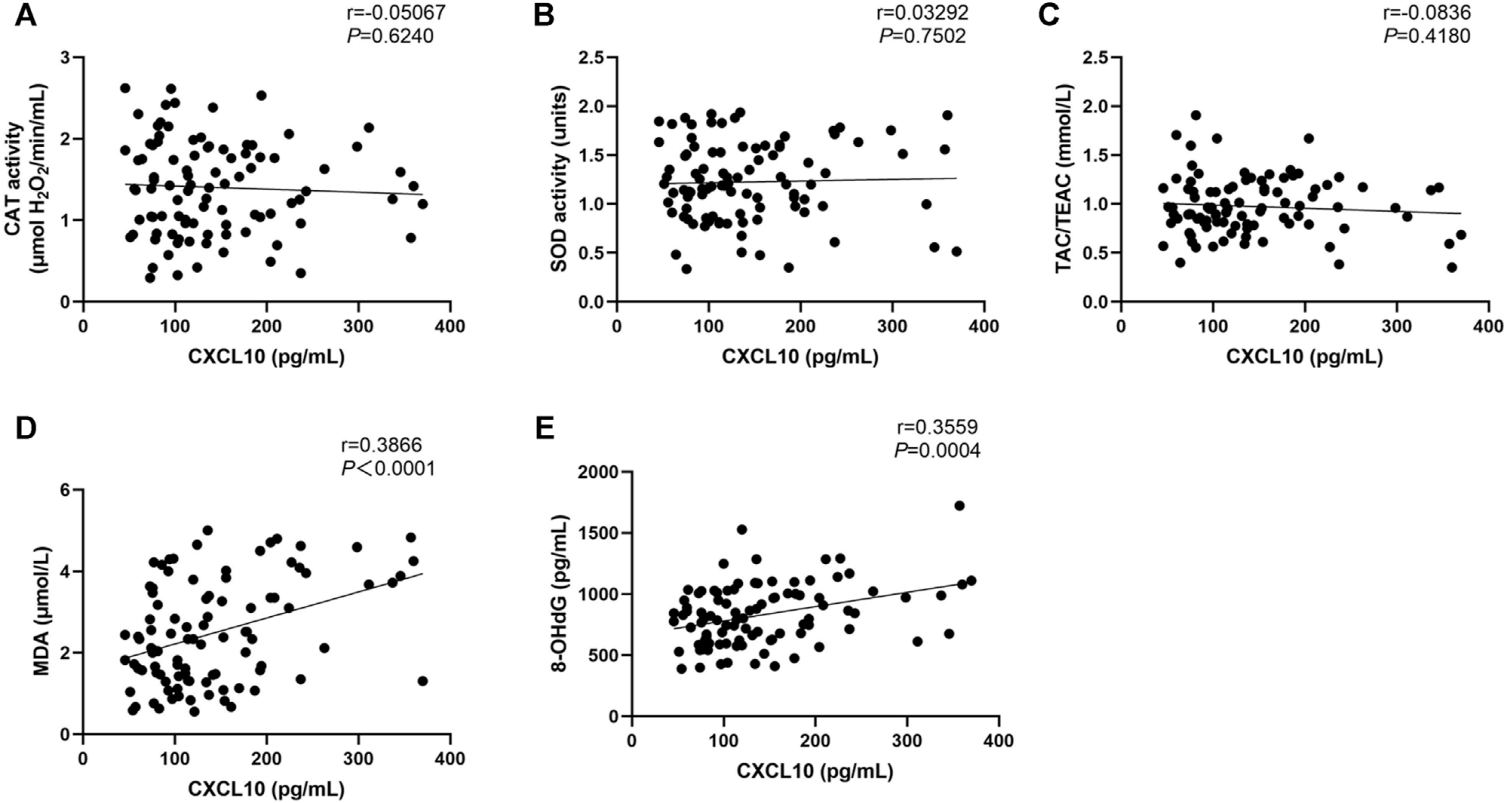
Correlation between CAT **(A)**, SOD **(B)**, TAC **(C)**, MDA **(D)**, and 8-OHdG **(E)** levels with the CXCL10 expression in NSV patients. CAT, catalase; SOD, superoxide dismutase; TAC, total antioxidant capacity; TEAC, Trolox equivalent antioxidant capacity; MDA, malondialdehyde; 8-OHdG, 8-hydroxy-2-deoxyguanosine.

Regarding physician-assessed disease activity, the serum levels of MDA and 8-OHdG were significant different among the three active stages of patients with NSV, whereas no significant CAT activity, SOD activity, or TAC levels were observed among these groups (Table 2). Serum CXCL10 has been confirmed as a novel biomarker in monitoring disease activity and guiding treatment of progressive vitiligo (Wang et al., 2016). We found no correlation between CXCL10 and CAT, SOD, or TAC (Figures 2A–C) but significantly positive correlation between CXCL10 and MDA (r = 0.3866, *p* < 0.0001) as well as 8-OHdG (r = 0.3559, *p* = 0.0004) (Figures 2D,E).

In patients who received treatment [narrow bound ultraviolet B light (NB-UVB) phototherapy, oral Chinese medicine, and topical corticosteroid/tacrolimus/pimecrolimus, alone or combined with each other] in the past 1 year, TAC levels were markedly higher compared with all other patients with NSV (Table 4), and both the MDA and 8-OHdG levels were significantly decreased in patients applying treatment relative to patients who received no therapy (Table 4). However, the CAT and SOD activity displayed no significant difference between the two groups (Table 4).

**TABLE 4:** Serum levels of OS markers in NSV patients receiving or without therapy in the past 1 year.

Groups parameter	Patients with NSV receiving treatment		*P*-value^a^
	No (n = 22), median(IQR)	Yes (n = 74), median(IQR)	
CAT activity (μmol H_2_O_2_/min/ml)	1.169(0.779–1.918)	1.423(1.006–1.874)	0.3614
SOD activity (units)	1.162(0.919–1.561)	1.195(0.914–1.584)	0.651
TAC/TEAC (mmol/L)	0.792(0.587–1.129)	0.964(0.844–1.169)	0.0082
MDA (μmol/L)	3.333(1.956–4.331)	2.103(1.304–3.333)	0.0018
8-OHdG (pg/ml)	946.3(707.7–1259)	794.9(608.8–976.6)	0.02

Abbreviations. OS, oxidative stress; NSV, non-segmental vitiligo; IQR, interquartile range; CAT, catalase; SOD, superoxide dismutase; TAC, total antioxidant capacity; TEAC, trolox equivalent antioxidant capacity; MDA, malondialdehyde; 8-OHdG, 8-hydroxy-2-deoxyguanosine.

a Two-tailed Mann–Whitney U test.

Further in multivariate logistic regression models, the association between serum OS markers including TAC and MDA with disease activity was significantly influenced by the vitiligo subtypes but not by the disease duration or the administration of therapy in the past 1 year (Table 5). After ruling out the effect of subtypes, there was no significant correlation between serum MDA levels and disease activity, but a clear association between serum TAC levels with disease activity was shown (Table 5). In contrast, serum 8-OHdG levels were not influenced by vitiligo subtypes, disease duration or the therapy, and remained significantly associated with disease activity (Table 5).

**TABLE 5:** Multivariate models investigating the association of TAC, MDA, 8-OHdG, and disease activity.

Independent variable	Coefficient	Wald test	OR(95% CI)	*P-*value
Model 1				
Duration	−0.001	0.506	0.999(0.995–1.002)	0.477
Subtypes	0.707	6.843	2.028(1.194–3.442)	0.009
Treatment	0.432	0.746	1.540(0.578–4.100)	0.388
TAC	−1.897	7.957	0.150(0.040–0.560)	0.005
Model 2				
Duration	−0.001	0.416	0.999(0.995–1.002)	0.519
Subtypes	0.636	5.682	1.889(1.120–3.184)	0.017
Treatment	0.08	0.028	1.083(0.424–2.768)	0.867
MDA	−0.005	0.607	0.995(0.981–1.008)	0.436
Model 3				
Duration	0	0.065	1.000(0.996–1.003)	0.798
Subtypes	0.471	2.974	1.602(0.938–2.735)	0.085
Treatment	0.808	2.357	2.243(0.799–6.303)	0.125
8-OHdG	0.003	11.363	1.003(1.001–1.005)	0.001

Abbreviations. OR, odd ratio; CI, confidence interval; TAC, total antioxidant capacity; MDA, malondialdehyde; 8-OHdG, 8-hydroxy-2-deoxyguanosine.

### OS Markers Were Associated With Disease Severity in Patients With NSV

**FIGURE 3:**
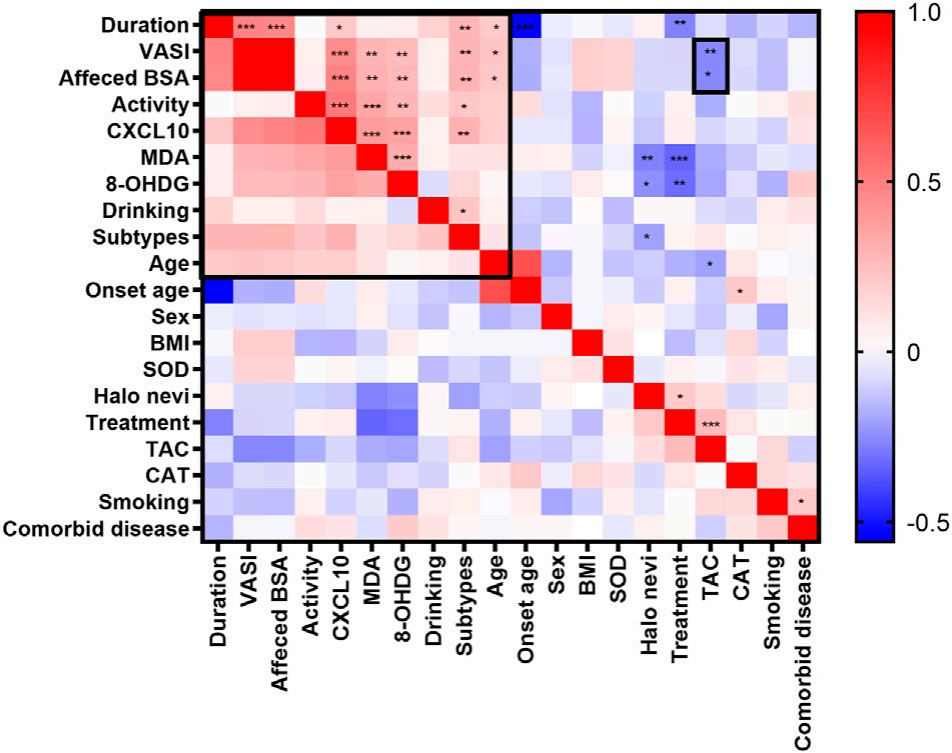
The correlation heatmap of clinical characteristics and serum OS markers by using Pearson correlations. The heatmap showing the positive (red) or negative (blue) correlations of all parameters with color intensity reflecting the strength of the correlation (−0.56 to +1). OS, oxidative stress. *p < 0.05; **p < 0.01; ***p < 0.001.

**FIGURE 4:**
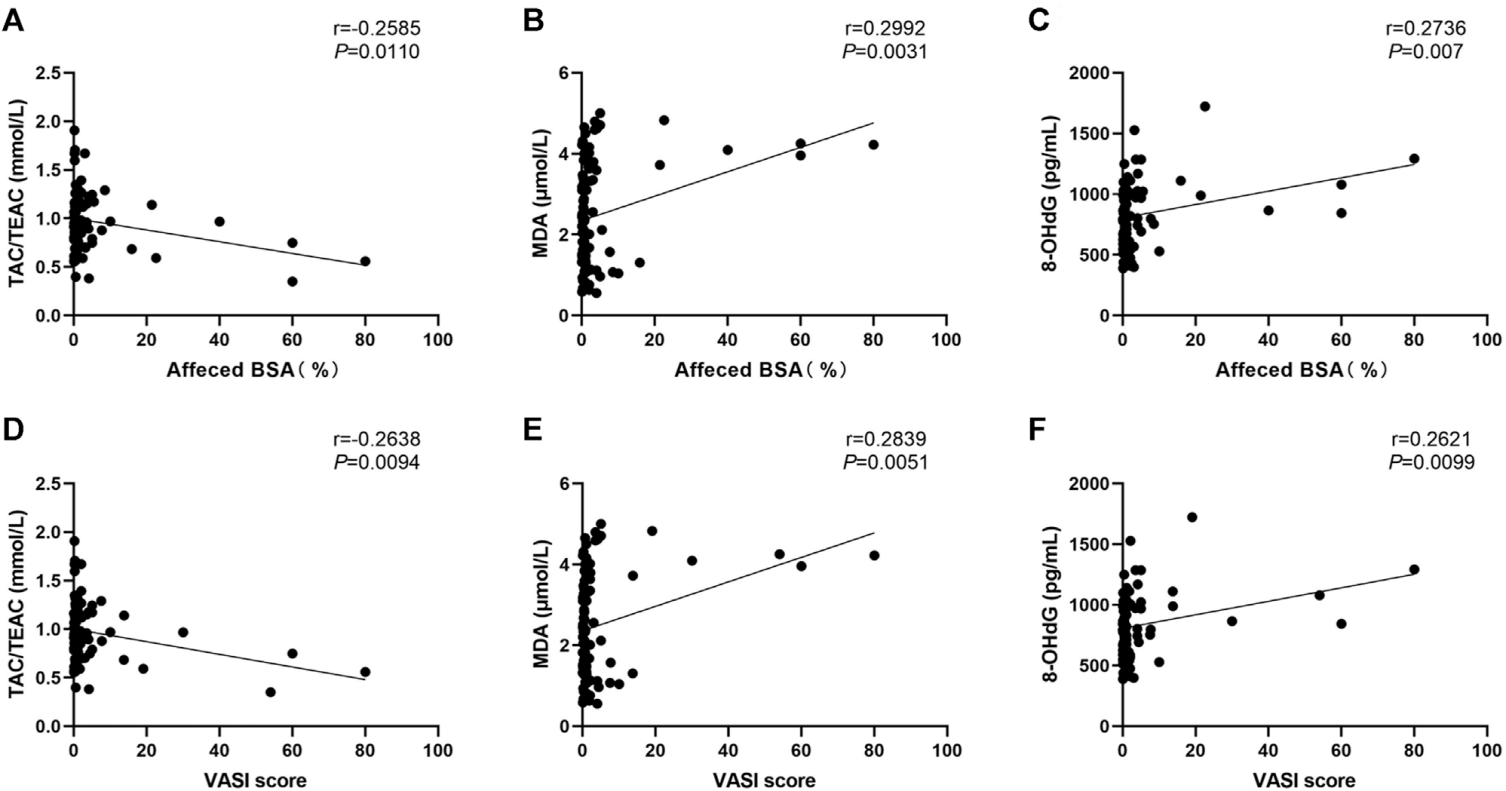
Correlation between serum TAC, MDA, and 8-OHdG levels with the affected BSA **(A, B, C)** and VASI score **(D, E, F)** in patients with NSV. TAC, total antioxidant capacity; TEAC, Trolox equivalent antioxidant capacity; MDA, malondialdehyde; 8-OHdG, 8-hydroxy-2-deoxyguanosine; BSA, body surface area; VASI, Vitiligo Area Scoring Index.

We also assessed how different characteristics, including sex, age, onset age, duration, subtypes, disease severity (as determined by using affected BSA and VASI scores), presence of Halo nevi, and associated comorbid diseases, relate to serum levels of OS markers. The correlation heatmap of all parameters were performed using Pearson correlations as shown in Figure 3 (red, positive correlation; blue, negative correlation; star signs display significant correlations). Selected individual scatter plots are also presented: Serum TAC was correlated with decreased affected BSA in NSV patients (r = −0.2585, *p* = 0.0110) (Figure 4A), and serum MDA and 8-OHdG levels correlated with an increased affected BSA in NSV patients (MDA, r = 0.2992, *p* = 0.0031; 8-OHdG, r = 0.2736, *p* = 0.0070) (Figures 4B,C). Similar correlations were found between VASI scores and serum TAC, MDA, and 8-OHdG levels in these NSV patients (TAC, r = −0.2638, *p* = 0.0094; MDA, r = 0.2839, *p* = 0.0051; 8-OHdG, r = 0.2621, *p* = 0.0099) (Figures 4D–F). No correlation between affected BSA or VASI scores and CAT or SOD serum levels could be shown in NSV patients (Figure 3). Importantly, in our study, we found a significant correlation between NSV subtypes and duration, VASI, affected BSA, disease activity, and CXCL10 (Figure 3), suggesting subtyping indeed influences the severity and activity of patients with NSV.

## Discussion

This study demonstrates lower levels of antioxidative capacity and higher values of OS damage markers in the serum of patients with NSV and points to their beneficial use as indicators of disease activity and severity. In this study, decreased serum CAT and SOD activity as well as TAC were found in patients with NSV, indicating an impairment of antioxidative capacity in vitiligo. It is worth noting that previous studies have observed controversial serum CAT activity in vitiligo patients (Deo et al., 2013), although most studies have observed reduced CAT activity in vitiligo (Agrawal et al., 2014; Ozel Turkcu et al., 2014). Genetic variants in the *CAT* gene, including *CAT* -89A/T, -262G/A, and -262T/C in the promoter region and -20T/C in 5ʹ-untranslated region, have detrimental effects on the expression or function of the enzyme (Forsberg et al., 2001; Bastaki et al., 2006; Komina et al., 2012; Kodydkova et al., 2014). We and other groups have demonstrated that the *CAT* -89A/T variants are associated with a significant decrease in CAT activity and a genetic predisposition for vitiligo in both Chinese and Indian population, especially in active and generalized vitiligo patients (Liu et al., 2010; Mansuri et al., 2017). Whereas, in Indian patients, the *CAT* –262G/A variant showed no change in CAT activity or risk of vitiligo (Mansuri et al., 2017). In addition, in Northwestern Mexicans, although the serum CAT activity was lower in vitiligo patients, they showed no association with any vitiligo clinical characteristics neither with their gene polymorphisms (Ochoa-Ramirez et al., 2019). These studies suggest that controversial results regarding the CAT activity in vitiligo patients seems to be at least partially related to the CAT polymorphisms and the role of ethnicity. For SOD activity in vitiligo patients, our results were consistent with the previous studies showing lower SOD activity (Koca et al., 2004; Khan et al., 2009); however, another studies reported SOD activity of vitiligo patient were increased compared with controls (Ozel Turkcu et al., 2014; Speeckaert et al., 2018). It has been reported that the change of SOD activity may be caused by SOD gene polymorphisms (Laddha et al., 2013). In addition to the genetic factors, the inconsistency is possibly due to the different sample size or patients as well as the diverse detection methods among different groups. Besides the antioxidative markers, we found highly increased OS damage markers including MDA and 8-OHdG in patients with NSV, which was consistent with our and other groups’ previous studies (Wei et al., 2013; Azzazi et al., 2021).

Our ROC analyses showed that the serum CAT, MDA, and 8-OHdG had a predictive capacity on diagnosis of the vitiligo. However, the sensitivity of using serum CAT or MDA cutoff values for vitiligo diagnosis was relatively low, about 45% and 32%, respectively. The combination of these OS indicators may improve the diagnostic sensitivity. Whereas, by using the cutoff values of serum 8-OHdG, about 80% of the patients with NSV can be identified with an acceptable false-positive rate of about 31%. Although there is considerable overlap between patients with NSV and HC, the difference of actual values of serum 8-OHdG between the patients group and HC group is remarkable, indicating that the diagnosis of NSV based on model with only one predictor is promising. Future studies with consecutive blood sampling are likely to display even better results by taking into account individual differences in baseline serum OS levels in patients with NSV.

This study describes the potential use of MDA and 8-OHdG as disease activity markers in patients with NSV. A study from Eastern India with a relatively small sample size also has showed the possibility of serum MDA to assess disease activity in vitiligo (Mukhopadhyay et al., 2019). Importantly, our results showed that there was a significant correlation between CXCL10 and MDA as well as 8-OHdG. CXCL10, as a novel serum marker for vitiligo activity, plays a critical role in mediating CD8^+^ T cells skin trafficking, thus promoting the disease progression and maintenance of depigmentation (Rashighi et al., 2014). Although CXCL10 is an interferon-γ–induced chemokine, our recent study has shown that, during OS, CXCL10 could also be significantly induced from keratinocytes to increase cutaneous T-cell immune response in patients with NSV (Li et al., 2020). Our results suggested that MDA and 8-OHdG, more than just being bystander products from environmental stimulus and OS damage markers for vitiligo progression, may also contribute to the inflammatory processes in the pathogenesis of NSV, which need to be further elucidated. Considering the effect of subtypes on association between serum OS markers and disease activity, serum TAC may also be a potential marker for disease activity but not MDA, which also need further study.

Furthermore, the relatively higher TAC and lower levels of MDA and 8-OHdG were observed in patients with NSV receiving treatment during the past 1 year, suggesting that conventional treatments including NB-UVB phototherapy, oral Chinese medicine, and topical corticosteroid/tacrolimus/pimecrolimus, alone or combined with each other could decrease the oxidative damage and enhance the antioxidant capacity. Because there is no significant change in CAT and SOD activity between the patients receiving treatment or without treatment, we might be able to consider the therapy to increase the activity of antioxidant enzymes in vitiligo treatment.

In addition, to accurately assess disease activity, precisely describing the severity of vitiligo is also crucial for treatment selection and prognostication. In our study, we found that serum TAC, MDA, and 8-OHdG levels correlated strongly with the affected BSA and VASI score in patients with NSV, whatever the patient at active stage or stable stage, with treatment or no treatment during past 1 year. These findings provided candidate serum OS markers that could be used to assess the severity of NSV. However, it is important to note that we used affected BSA and VASI scores to assess the disease severity, which is subjected to inter and intra-assessor variability because such scores require dermatologists to estimate the area of affected lesion and the degree of depigmentation (Komen et al., 2015; van Geel et al., 2016; van Geel et al., 2017). A more accurate and reliable evaluation method for disease severity needs to be studied, so as to further verify the potential of the candidate serum markers as disease severity indicators.

It is also noteworthy that the subtypes of NSV could affect the relationship between serum OS levels and disease activity and severity, although the 8-OHdG levels remained significant with disease activity in our study. The sample sizes of patients with certain subtypes were too small to further explore the clinical significance of these serum OS markers for disease activity or severity assessment in specific subtypes. Similar unbalanced sampling also existed in terms of the treatment history of the patients. Further study with larger sample size of both NSV patients and HC, more balanced enrollment of various subtypes, treatment history, and anatomical sites of skin lesion, and other potential confounders may provide a better interpretation.

In conclusion, this study shows decreased CAT, SOD, and TAC and increased MDA and 8-OHdG serum levels in patients with NSV. The clear correlations of both MDA and 8-OHdG serum levels with disease activity, affected BSA, and VASI scores shed light on the value of these markers in detecting vitiligo activity and severity in patients with NSV. Future multicenter studies with larger sample size and consecutive blood sampling are needed to determine the cutoff serum levels for their final usefulness as biomarkers in clinical practice.

## Data Availability

The original contributions presented in the study are included in the article/Supplementary Material, further inquiries can be directed to the corresponding author.
